# Entrepreneurship, Corporate Social Responsibilities, and Innovation Impact on Banks’ Financial Performance

**DOI:** 10.3389/fpsyg.2021.680661

**Published:** 2021-08-25

**Authors:** Jianhua Wei, Rong Xiong, Marria Hassan, Alaa Mohamd Shoukry, Fares Fawzi Aldeek, J. A. Khader

**Affiliations:** ^1^Department of Business Administration, Handan Economic and Technological Development Zone, School of Management Engineering and Business, Hebei University of Engineering, Handan, China; ^2^Department of Business Administration, Rajamangala University of Technology Krungthep, Bangkok, Thailand; ^3^Department of Management Sciences, Islamia University of Bahawalpur, Bahawalpurm, Pakistan; ^4^Arriyadh Community College, King Saud University, Riyadh, Saudi Arabia; ^5^Administrative Sciences Department, Community College, King Saud University, Riyadh, Saudi Arabia; ^6^College of Business Administration, King Saud University - Muzahimiyah Branch, Al-Muzahmiya, Saudi Arabia

**Keywords:** entrepreneurship, return on assets, corporate social responsibilities, innovation, return on equity, board size, financial performance

## Abstract

The basic aim of this research was to check the impact of innovation, corporate social responsibilities (CSR), and entrepreneurship on the monetary performance of banks in five different countries: Qatar, Pakistan, China, the United States (US), and France. This research was conducted to measure the relationship of these factors and innovative workforce activities. The secondary data were collected from websites of twenty five banks in different countries, including Islamic and conventional banks. Different econometric analyses, such as descriptive statistical analysis, correlation coefficient test for measuring the interaction, and ordinary least square regression analysis for determining the impact of dependent and independent variables, were carried out. In the present study, entrepreneurship, CSR, and innovation were taken as independent variables. Board size, frequency of assemblies, and self-employed with large shareholders were included as sub-parts of entrepreneurship. On the other hand, the financial performance of banks was taken as the dependent variable. Return on assets (ROA) and return on equity (ROE) were considered parts of economic performance. The overall conclusions drawn in this study showed that there was a significant relationship between all the studied variables. The research provided useful insights into the long-debated question regarding the relevance of entrepreneurship and CSR.

## Introduction

Entrepreneurship is important due to the departure of control and ownership in openly held organizations. It encompasses a collection of skills in processes, proper mechanisms, and relations used by different parties in the corporations. It is also a combination of board and committee, all policies, legalities, rules and regulations system, overall hierarchy, and internal control (Kojima et al., 2017).

Entrepreneurship and its components identify all rights among participants including managers, auditors, creditors, the board of directors, and other shareholders (Chen and Yu, 2017). Corporate entrepreneurs are very important for organizations because they increase the possibility of recovering from conflicts between upper management and shareholders. Entrepreneurship is a scheme through which organizations are measured and monitored. The Board of Directors are mainly accountable for the success of corporations. The role of stockholders in entrepreneurships is to assign the auditors and directors to satisfy themselves and to provide a suitable overall governance structure (Al-ahdal et al., 2020).

Sometimes entrepreneurship is broken down into four elements, also called the four Ps institutions: people, purpose, process, and performance (Wondem and Batra, 2019). These philosophies provide guidelines about entrepreneurship on why entrepreneurship exists, the governance mechanism, and how it works and operates. Large shareholders influence the management of a company. They also have the power to shape the company’s investment decisions and deploy minority shareholders’ resources. The block holder often acts as an agent who controls the principal’s resources (i.e., minority shareholders) (Choi and Park, 2019). In this way, entrepreneurship shapes the relationship between large and small self-employed shareholders by providing minorities the means to safeguard their interests, if they are different from those of the majority shareholders. The misalignment between the managers and shareholders’ risk tolerance might be damaging for shareholders since it can result in an allocation of resources that is not efficient from a shareholder’s point of view.

To sum up, the principal-agent problem arises in modern corporations because of the coexistence of four elements:

• Agent’s self-interest.
• Division of ownership and control.
• Information asymmetry between agent and principal.
• Residual decision rights allocated to the agent.

Skill is involved in improving the operation and creating more efficiency, mitigating risk, enhancing the access to capital, and providing a defense to stakeholders (Mansur and Tangl, 2018). An improved operation also produces more accountability and transparency for builders and investors. Malik Riaz, Bill Gates, and Larry Page are examples of good entrepreneurs. They usually outperform other firms (Ahmad et al., 2019). The main focus of entrepreneurship is to support the investor and help to finance further growth. The entrepreneurship assessment used in this work directly or indirectly evaluates all these methods. Apart from these three governance-related mechanisms, the research study includes the entrepreneurship instruments and the financial control performed by external auditors. The enforcement of these instruments is paramount for companies (Sarpong-Danquah et al., 2018).

Corporate social responsibilities (CSR) also play an important role in the firm’s efficacy. CSR shows the organization’s self-accountability related to the social as well as environmental concerns. The organizations that put their attention toward the social and environmental responsibilities also put extensive attention toward their efficiency and strive for high financial performance (Ali et al., 2017). CSR are effective regulations on social and environmental matters that must be followed by organizations to survive in society and avoid environmental degradation to enhance the social norms (Chen et al., 2018). CSR restrict the ability of organizations to damage societal norms and the environment by providing the framework of responsibilities that must be performed by the organization. Thus, CSR is a necessary element for the organization to survive in the society and to provide a positive impact on the firm’s efficiency. Therefore, the present study considered CSR to examine the firm’s financial performance.

Innovation is about creating new value in a new way. Basically, innovation is a development, creation, and implementation process related to new products and services with improved efficiency, competitive advantages, and increased effectiveness in organization-related work (Akram et al., 2011; Nawaz et al., 2019).

This research study’s main emphasis was to measure the association of entrepreneurship, CSR, and innovation with the economic performance of banking sectors. Entrepreneurs play an essential role in every corporation. For this research we used the number of members on the board of management, the frequency of meetings, and number of large stakeholders as independent variables. These are all the subunits of entrepreneurship. Process innovation and product innovation along with CSR were also considered independent variables for determining the impact of financial performance.

The emphasis of this research was to examine the extent of relation amid firm-specific entrepreneurship practices and firms’ actual routines. We tried to provide an answer to the long-debated question, “Does entrepreneurship have a substantial impact on a firm’s results?”

Financial performance is an essential subjective measure that measures a firm’s performance and considers how well a firm used financial assets from the different primary modes of all businesses and generates revenues. There are several performance indicators related to financial performance i.e., revenue, profit margin, client retention rate, and average class attendance to increase the probability of measuring productivity (Srivastava and Bhatia, 2020). Similarly, five types of financial statements show financial performance, such as financial position, statement of cash flow, equity statement, income statement, and financial information. In this research data were collected from these annual financial statements.

### Research Questions

• How do entrepreneurship, CSR, and innovation affect financial performance in banking sectors?
• How can financial performance of banks be measured?

## Literature Review

Al-Rahahleh (2017) conducted a study on entrepreneurship in corporation and bank’s financial performance. This research was conducted in the Arabian Peninsula. This study used different variables related to entrepreneurship, such as board size and bank age. The sample used in this research comprised both Islamic banks and conventional banks that operate in seven Arabian Peninsula countries including the Kingdom of Saudi Arabia, Oman, Yemen, and Qatar. This research’s findings showed that there was an important association between board size, bank age, and bank’s financial performance. Overall results described a meaningful association between entrepreneur and financial performance. The studies of Matousek and Tzeremes (2016) have implied that the technical efficiency of the banks can be measured using two measures: Data Envelopment Approach (DEA) and Free Disposal Hall (FDH). Djebali and Zaghdoudi (2020) evaluated entrepreneur performance testing the entrepreneurship performance in relation to Tunisian banks based on the specific GMM system analysis. This study’s main aim was to examine the internal entrepreneur impact on banking sectors and their performance. For this purpose, they used annual data from 10 Tunisian banks registered in the stock exchange of Tunisia. Data were used from between 1998 and 2015. The results indicated that the correlation between the state’s presence, the attendance of ID (Independent directors), and the board of directors had an optimistic and significant association. On the other hand, some CEO compensation and institutional and foreign stakeholders represent a negative consequence on the presentation of banking sectors. Banking performance is heavily affected by return on assets (ROA) and number of shareholders (Fukuyama and Matousek, 2017). Owiredu and Kwakye (2020) investigated the impression of entrepreneurship on commercial banks’ economic performance in Ghana. Data for this research were composed from the yearly reports and the financial statements of selected banks from 2007 to 2016. For this purpose, they used different models, including the random sampling model, the ordinary Regression analysis, and the OLS model, for analysis. Results revealed an important and optimistic association between entrepreneurship and a bank’s financial performance.

Social corporate responsibilities (CSR) also has a positive effect on the financial performance of an organization because it improves the efficiency of the organization not only in terms of social and environmental concerns but also with achieving the high performance goals of the organization. In many previous studies it was revealed that banking is highly affected by shareholders (Farrukh et al., 2017; Dakhlallh et al., 2019). They also enhance the efficiency to achieve the goal of maximization of shareholders’ wealth (Beck et al., 2018). In addition, CSR are effective regulations related to the social norms which improve the efficiency of an organization toward social concerns along with the financial performance of an organization (Fernández-Gago et al., 2018). Moreover, the banks that implement CSR within an organization are more efficient and high-performance-oriented than banks which are not effectively implementing CSR in their organization (Jain et al., 2015). Thus, CSR are necessary elements for an organization and they have a positive impact on a firm’s efficiency. Therefore, in the present study we took this into consideration and examined the role of CSR for firm financial performance.

## Materials and Methods

### Research Method

This study’s basic theme was to inspect the influence of entrepreneurship, CSR, and innovation on the economic presentation in banking sectors. To achieve this objective, we used data from five countries: Pakistan, Qatar, China, France, and the United States. Twenty-five banking sectors, both Islamic and conventional, were selected for empirical study in every country.

### Board of Directors

The board of managements, the leading authority concerned with manager monitoring, is appointed by the shareholders and acts on their behalf to monitor the managers’ decision-making activities to ensure their good faith and their shareholder value creation attitude (Qadorah and Fadzil, 2018). Directors participate in the business’s economic life and have the responsibility of accountability to monitor the managers’ actions and rectification (Jizi, 2017). They may or may not hold executive roles within the organization. Managers must report periodically to the Board of Directors and the latter have to evaluate the proposals and approve them (Miyajima et al., 2018).

### Corporate Social Responsibilities

Corporate social responsibilities (CSR) are the responsibilities related to the social norms and environmental concerns that must be implemented by the organization to protect the society and environment. The organizations that implemented and followed CSR are considered more efficient not only toward societal norms and environmental concerns but also toward attaining high financial performance (Asmeri et al., 2017). Thus, the organizations that have implemented and followed CSR has been assigned the value “1” while the organizations that have not implemented and followed CSR have been assigned the value “0.”

Outsiders rely much more on objective evaluation criteria given their lack of understanding of businesses and firm practices, thus enabling ex-post financial controls (evaluating the outcome of manager’s conduct). On the other hand, insiders adopt more subjective criteria, based on business and firm knowledge that descends from their past experiences. Hence, insiders can establish ex-ante strategic controls on managers’ decisions (evaluating their behavior).

### Large Shareholder Rights

The division of powers and the unfeasibility of complete contracts have awarded managers great authority over the company’s life. However, in light of their owner’s role, corporate laws provide shareholders with the power to have a say in the company’s management. This power is fragmented into minor separate rights which shareholders can enforce against executives and managers. The existence of a set of rights awarded to every shareholder indiscriminately, proportionally to the size of their stake, aims at ensuring not only the protection of owners against their agents, e.g., managers, but also against larger controlling shareholders who can exert tremendous pressure on management to pursue their private interests, which might be different from those of minorities. The exercise of these rights can protect themselves from managerial misbehavior and the supremacy of large block holders. Every national legal framework prescribes a shareholder meeting which is called to rectify some resolutions of primary importance for the company. These matters are crucial for the company’s life i.e., mergers and acquisitions, financial statement approval, the election of the board members, and so forth. To this extent, shareholder rights are characteristics of a given legal framework rather than of a single company. However, the company can take some legal measures that indirectly impede the exercise of these rights. These could include takeover defenses that might entrench management or the introduction of misalignment between ownership and voting power in the bylaws (Hansen and Block, 2020).

### External Auditor

Audits embed all the activities that are undertaken to examine and verify the company’s records and statements. In past years, external audits have attracted attention due to the occurrence of scandals regarding the independence and good faith of external auditors like the Enron scandal, which eventually led to the company’s bankruptcy. External auditors exercise a gate keeping role since they provide an independent judgment and assure the market that the company’s financial condition is portrayed truthfully. External audits reduce the agency problem by relying on independent and objective supervision performed by competent authorities without any linkage to the organization. Research analysis includes the external audit as an additional entrepreneurship instrument to follow the categorization proposed by Institutional Shareholder Services (Owen and Temesvary, 2018).

In this research paper, the effectiveness of all the above-described tools were evaluated to grade entrepreneurship. However, these were classified differently. In most developed markets, entrepreneur systems have reached a high level of development. Companies can attract reasonable capital amounts by investors ensuring that their money will be deployed in their best interests. The mitigation of the agency problems and the subsequent allocation of responsibilities allow shareholders to “trust” the company. Indeed, the poor economic results of any company are not entirely dependent on the existence of managers’ good faith. They can well act to satisfy their shareholders but can still take wrong decisions that diminish owners’ wealth. The organization operates in a competitive field and the uncertainty which is systemically associated with its actions creates risks to shareholders. Entrepreneurship assures the shareholders that the rewards they receive for their residual claim are the outcome of a set of informed decisions taken in their best interests (Wahyudin and Solikhah, 2017). Their finance provider role has to be rewarded in light of their investments which are essential to allow the corporation to grow (Khan et al., 2013).

### Sampling

This research paper describes the inspiration of entrepreneurship, CSR, and innovation on a bank’s economic performance. The sample size of this research was selected from five countries: Pakistan, China, Qatar, France, and the United States. The statistics were collected from the bank’s annual and financial information by selected banks of these countries including Islamic banks and conventional banks.

### Hypothesis Development

H0 = There is no association between entrepreneurship, CSR, and innovation on the financial performance of banking sectors.

H1 = There is an important association among board size and financial performance.

H2 = There is an associated impact of incidence of meetings and the bank’s financial performance.

H3 = There is a specific association among entrepreneurship and financial performance.

H4 = The number of large shareholders shows an optimistic and important consequence on a bank’s financial performance.

H5 = CSR has a significant relation with financial performance.

H6 = The innovation has a significant relationship with financial performance and entrepreneurship.

Entrepreneurial instruments, such as performance-based executive compensation, are aimed at making opportunistic behavior financially unattractive for managers. The monitoring performed by the Board of Directors ensures an adequate evaluation of managerial conduct and impedes the occurrence of shareholder value-destroying actions.

In the presence of proper control, the incentive that managers have in expropriating bank’s resources drops since the probability of their discovered malfeasances increases. In this situation, the management team would be replaced, which is highly undesirable for managers. Furthermore, the Board of Directors’ impartial and objective control is necessary to ensure minority shareholders that a controlling shareholder’s presence would not diminish their returns.

**FIGURE 1:**
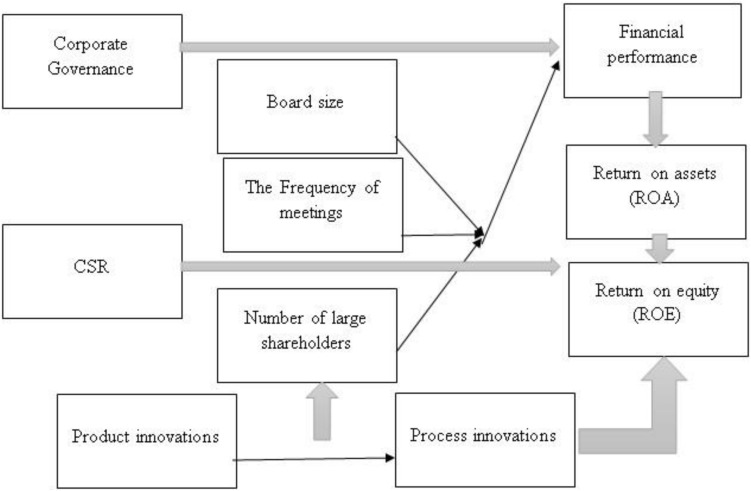
Theoretical framework.

Figure 1 shows the theoretical frameworks and Table 1 shows the variables. Connection between Entrepreneurship, CSR, and Performance: The second set of models investigated the relationship between the performance indicators and scores awarded to each bank for each of the three entrepreneurship pillars. Again, the performance indicators were dependent variables and the overall score was the regressor.

**TABLE 1:** Variables.

Sr. no	Variables	Notation
	Independent variable	
1	Entrepreneurship	CG
2	Corporate social responsibilities	CSR
3	No of the large shareholders	NLS
4	Frequency of meetings	FOM
5	Board size	BS
6	Innovation	I
7	Product innovation	PI
8	Process Innovation	PI
9	Dependent variable	DV
0	Financial performance	FP
11	Return on assets	ROA
12	Return on equity	ROE

The two equations were:

$$\begin{array}{c} MODEL2.1:ROE_{it}=\beta_{0}+\beta_{1}BS_{it}+\beta_{2}{\text{FOM}}_{it}+\beta_{3}{\text{CSR}}_{it}\\ +\beta_{3}{\text{NOLS}}_{it}+\varepsilon \end{array}$$

$$\begin{array}{c} MODEL2.1:ROA_{it}=\beta_{0}+\beta_{1}BS_{it}+\beta_{2}{\text{FOM}}_{it}+\beta_{3}{\text{CSR}}_{it}\\ +\beta_{4}{\text{NOLS}}_{it}+\varepsilon \end{array}$$

## Results

Table 2 describes the descriptive statistical analysis of all variables: board size; the frequency of meetings; number of large shareholders; CSR; the ROA; and return on equity (ROE) by the value of the mean, standard deviation, medium value, maximum values, and minimum values. Entrepreneurship, CSR, and innovation were considered as independent variables and financial performance was the dependent variable. Return on assets and returns on equity were sub variables related to financial performance. Their mean values were 25.422 and 0.518, respectively. The median values were 0.43 and 18.32. Their standard deviation values were 0.279 and 16.482 which showed that “ROA” and “ROE” deviated 27 and 16% from their means. Similarly, the return on asset’s maximum value was 0.990 and the ROE’s maximum value was 93.32. This statistical analysis used 275 observations for measuring the influence between entrepreneurship and innovation on the bank’s financial performance. The probability value was put at 0.000 which showed a 100% significance level. The skewness values were 0.49, 0.07, 1.47, 0.29, and 1.56, respectively, for all the variables. Results described the overall relationship between them.

**TABLE 2:** Descriptive statistical analysis.

	Board size	Frequency of meetings	Number of large shareholders	Return on assets	Return on equity	CSR
Mean	13.68364	5.578182	24.43445	0.518331	25.42273	0.532112
Median	13.00000	6.000000	18.01000	0.430000	18.32000	0.522513
Maximum	20.00000	9.000000	76.32000	0.990000	93.32000	1
Minimum	7.000000	3.000000	9.310000	0.110000	9.310000	0
Std. dev.	2.750897	1.141566	14.89482	0.279440	16.48223	0.314752
Skewness	0.492071	0.079864	1.471600	0.295661	1.562766	0.084511
Kurtosis	2.416709	2.534174	4.350983	1.687623	4.890914	2.314854
Jarque-Bera	14.99626	2.778721	120.1702	23.74159	152.9058	2.935612
Probability	0.000554	0.249235	0.000000	0.000007	0.000000	0.000000
Observations	275	275	275	275	275	275

Table 3 represents the correlation coefficient analysis between entrepreneurship, CSR, and financial performance. The correlation coefficient examination explained the inter-correlation among variables, such as board size and ROA which showed a negative relationship between them at –0.1821. The frequency of meetings showed a positive relationship with ROA, 0.0663, and a negative relationship with ROE, –0.069. Return on equity showed a positive relationship with board size at a rate of 0.120. CSR had a positive association with ROA, i.e., 0.04195, and ROE, i.e., 0.3176. The number of shareholders and ROA presented a positive relationship of 0.0503. One represents the 100% significance level among all variables, such as board size, number of shareholders, and occurrence of meetings, showed the entrepreneur performance regarding financial aspects. The frequency of meetings and ROA also described a positive relationship at a 0.066 level of significance. Board size and ROA showed an 18% significance level.

**TABLE 3:** Correlation coefficient.

	Board size	Frequency of meetings	Number of large shareholders	Return on assets	Return on equity	CSR
Board size	1	0.0572	–0.173	–0.182	0.120	0.321
Frequency of meetings	0.0572	1	–0.077	0.066	–0.069	0.015
Number of large shareholders	–0.173	–0.077	1	0.050	–0.137	0.329
Return on assets	–0.182	0.066	0.0503	1	–0.079	0.041
Return on equity	0.120	–0.069	–0.137	–0.079	1	0.317
CSR	0.321	0.0154	0.329	0.041	0.317	1

Table 4 explains the cross-section results related to ordinary least square regression analysis when the dependent variable was the ROA. The value of standard deviation, the T-statistic value, and the probability value described the relations between a dependent variable and independent variables. The board size was the independent variable and as a part of entrepreneurship, its coefficient value was –0.0179. Its standard deviation value was 0.0061, the t-statistic value was –2.9099, and the probability value was 0.003. Results showed that the board size presented a negative relationship but a significant connection with financial performance. The second independent variable was the frequency of meetings. Its coefficient value was 0.018. The standard deviation value was 0.0146. Its t-statistic value was 1.23 and the probability value was 0.216. The third predictor was the CSR; its coefficient value was 0.0325. The standard deviation value was 0.0121. Its t-statistic value was 2.679 and the probability value was 0.0210. It showed positive and significant association among CSR and ROA. Regression analysis indicated that the frequency of meetings showed positive linkage with the financial performance, but it was not significant. The number of shareholders was also taken as an independent variable and part of entrepreneurship. Its *t*-statistic value and coefficient value were 0.0003 and 0.3017, respectively. Its probability value was 0.76. The results showed that there was a positive but not significant connection with the financial performance of banking sectors. The value of R-square was 0.042 and its probability value was 0.0199 which showed 1.99% significant association. The F-statistic value was 2.97 and the adjusted R-square value was 0.027.

**TABLE 4:** Regression analysis: descriptions dependent variable is the return on assets.

Variable	Coefficient	Std. error	t-statistic	Probability
C	0.6763	0.1267	5.3357	0.000
Board size	–0.0179	0.0061	–2.9099	0.003
Frequency of meetings	0.0182	0.0146	1.2393	0.216
CSR	0.0325	0.0121	2.6793	0.021
Number of large shareholders	0.0003	0.0011	0.3017	0.763
The value of R-squared	0.0421			
Value of adjusted R-squared	0.0279			
Standard error of regression	0.2755			
The sum of squared residuals	20.493			
The log-likelihood	–33.167			
Value of F-statistic	2.9715			
Probability (F-statistic)	0.0199			

Table 5 also represented the ordinary least square regression analysis related to financial performance. In this table, ROE was considered dependent for measuring the financial performance of banking sectors and measuring the relations between entrepreneurship and innovation regarding financial performance. The total panel of observation was 275. The board size was an independent variable. Its coefficient value was 29.1862 and the standard deviation value was 7.67. Its value of t-statistic was 3.802 and its probability value was 0.002. Results showed that the board size represented an optimistic and most important link with the ROE at an 100% significance level. The frequency of meetings was also taken as part of entrepreneurship in a way that its coefficient and t-statistic values were –1.1672 and –1.244, respectively. It indicated that the negative relationship of its probability value was 0.17 showing no significance at a rate of 17%. CSR coefficient value was 0.1439. The standard deviation value was 0.0723. Its t-statistic value was 1.990 and the probability value was 0.0214. It showed positive and significant association among CSR and ROE. The number of large shareholders was also an independent variable and part of entrepreneurship. Its coefficient value was –0.1382 and the t-statistic value was –2.055, which showed a negative relationship with ROE. Still, it was significant because of its probability value: 0.04. The importance of R-square was 0.038. Overall probability value was 0.032. Its F-statistic value was 2.672. Overall results rejected the H0 (null hypothesis) and accepted all alternative hypotheses: H1, H2, and H3.

**TABLE 5:** Dependent variable: return on equity.

Variable	Coefficient	Std. error	t-statistic	Probability
C	29.18626	7.675065	3.802738	0.0002
Board size	0.561698	0.369508	1.520127	0.1296
Frequency of meetings	–1.167295	0.868014	–1.344788	0.1798
CSR	0.143921	0.072321	1.990031	0.0214
Number of large shareholders	–0.138232	0.067241	–2.055781	0.0408
The value of R-squared	0.038088			
Value of adjusted R-squared	0.023837			
Standard error of regression	16.28460			
The sum of squared residuals	71600.82			
The log-likelihood	–1154.996			
Value of F-statistic	2.672725			
Probability (F-statistic)	0.032478			

Table 6 explained the Pedroni co-integration test among all variables including entrepreneurship, CSR, innovation, and financial performance. Results represented the alternative hypothesis with statistic value, the value of probability, and observational values related to the legs and variances. These results accepted the alternative view and rejected the null hypothesis associated with entrepreneurship and financial performance.

**TABLE 6:** Hypothesis.

Alternative the hypothesis: common AR Coefficients (within-dimension)					

	Statistic value	Probability	Weighted Statistic value	Probability	
The panel v-statistic	–0.927719	0.8232	–1.165478	0.8781	
The panel rho-statistic	2.247529	0.9877	2.902417	0.9981	
The panel PP-statistic	–6.833293	0.0000	–3.158293	0.0008	
The panel ADF-statistic	–0.404606	0.3429	1.398021	0.9189	

**The alternative hypothesis: individual AR Coefficients (between-dimension)**					

	**Statistic value**	**Probability**			

Group rho-statistic	5.067267	1.0000			
Group PP-statistic	–6.057705	0.0000			
Group ADF-statistic	0.284462	0.6120			

**Overall cross-section specific results**					
	
**The Phillips-Peron results (non-parametric)**					

**Cross ID**	**AR(1)**	**Variance**	**HAC**	**Bandwidth**	**Observation**

1	–0.408	1.499276	0.339436	9.00	10
2	–0.111	4.828203	4.828203	0.00	10
3	–0.039	4.884368	4.884368	0.00	10
4	–0.222	7.435828	7.435828	0.00	10
5	–0.347	1.377512	0.719122	5.00	10
6	–0.237	1.003110	0.239579	9.00	10
7	–0.386	0.776175	0.547335	2.00	10
8	–0.484	1.443591	0.243868	8.00	10
9	0.192	0.373116	0.433681	1.00	10
10	–0.465	1.327791	1.354859	1.00	10
11	–0.155	2.023940	2.001785	1.00	10
12	–0.376	5.985166	6.180868	1.00	10
13	–0.485	5.902544	2.466254	7.00	10
14	–0.267	3.493007	3.765426	1.00	10
15	–0.170	2.184042	1.081399	7.00	10
16	0.436	3.075256	2.745813	2.00	10
17	–0.428	6.779533	6.779533	0.00	10
18	0.586	0.785261	1.127738	2.00	10
19	0.383	2.941114	2.941114	0.00	10
20	–0.392	7.541704	1.824278	9.00	10
21	–0.122	1.334927	1.334927	0.00	10
22	0.145	0.527217	0.634162	2.00	10
23	–0.203	1.833193	1.892224	1.00	10
24	–0.454	6.110362	2.159845	9.00	10

**Augmented Dickey-Fuller results (parametric)**					

**Cross ID**	**AR (1)**	**Value of variance**	**Total lag**	**The max lag**	**Observation**

1	–1.267	0.536633	1	–	9
2	–0.090	5.165431	1	–	9
3	–0.292	5.177026	1	–	9
4	–0.171	8.195278	1	–	9
5	–0.778	1.229689	1	–	9
6	–0.809	0.681627	1	–	9
7	–1.165	0.691117	1	–	9
8	–1.160	1.096966	1	–	9
9	0.146	0.333277	1	–	9
10	–0.576	1.312699	1	–	9
11	–0.251	2.232980	1	–	9
12	–0.262	6.578937	1	–	9
13	–1.261	1.973118	1	–	9
14	0.104	3.548082	1	–	9
15	–0.812	1.771987	1	–	9
16	0.103	2.385752	1	–	9
17	–0.583	5.936183	1	–	9
18	0.753	0.730156	1	–	9
19	0.191	3.179142	1	–	9
20	–1.016	6.133864	1	–	9
21	–0.181	1.468779	1	–	9
22	0.601	0.398824	1	–	9
23	–0.021	1.990258	1	–	9
24	–1.252	1.958642	1	–	9

Table 7 described the unit root test analysis among variables with statistics’ help. The total observations were 225 and the overall cross-section value was 25. This analysis used multiple tests related to the hypotheses. The Levin, Lin, and Chu *t*-test showed statistic value as –0.3167. Its probability value was 0.37. Other tests were Pesaran and Shin W-stat tests. It showed that statistic value was –1.03189. Its probability was 15%. The observed values of this analysis were 225. The ADF-Fisher Chi-square was another test related to the asymptotic Chi-square distribution. Its statistic value was 68.93 and its probability value was 0.0391. It showed a 3% significance level.

**TABLE 7:** Unit root test.

Methods test	Statistic	Probability**	Cross sections	Observation
**Null: the Unit root (assumes common unit root process)**				
Levin, Lin, and Chu t*	–0.31671	0.3757	25	225
**Null: unit root (assumes individual unit root process)**				
Im, Pesaran, and Shin W-stat	–1.03189	0.1511	25	225
ADF–Fisher Chi-square	68.9313	0.0391	25	225
PP–Fisher Chi-square	82.2652	0.0027	25	250

**The probabilities for Fisher tests are computed using an asymptotic Chi-square distribution. All the other tests assume asymptotically are normality. *Stationary at level.

Table 8 shows the correlation matrix has shown the links among the variables. The values highlighted no high linkage among the items while all the predictors have a positive association with the bank performance except numbers of large shareholders and CSR.

**TABLE 8:** Correlation matrix.

Variables	BS	FOM	NOLS	CSR	ROA
BS	1.000				
FOM	0.019	1.000			
NOLS	–0.114	0.193	1.000		
CSR	0.138	0.126	0.176	1.000	
ROA	0.061	–0.039	–0.190	–0.478	1.000

In Table 9, the VIF displays the multicollinearity in the model. The values show that no multicollinearity exists in the model because the VIF values are lower than five.

**TABLE 9:** Variance inflation factor.

	VIF	1/VIF
BS	1.641	0.609
FOM	1.454	0.688
NOLS	1.413	0.708
CSR	1.106	0.904
ROA	1.077	0.929

Table 10 examined the normality of the variables and values of the Skewness and Kurtosis, with the probability values less than 0.05, which indicates that there is a normality issue in the model.

**TABLE 10:** Skewness and kurtosis test.

	Pr(Skewness)	Pr(Kurtosis)	adj_chi2(2)	Prob > chi2
BS	0.000	0.000	.	0.000
FOM	0.000	0.000	55.840	0.000
NOLS	0.526	0.000	15.510	0.000
CSR	0.000	0.000	37.490	0.000
ROA	0.000	0.001	27.630	0.000

Table 11 explained the coefficient confidence interval of all variables including board size, the frequency of meetings, entrepreneur, CSR, and financial performance positions. This interval was divided into three parts at 90, 95, and 99% and showed the low level and high level of all variables. The board size coefficient value was 29.186. Its 99% interval low value was 9.275 and high-level value was 49.096. Similarly, the frequency of meetings’ value of the coefficient was 0.5616. Its low value showed a negative interval in every part as –2.599, –2.876, and –3.419, respectively. The coefficient value of ROA was –3.0108 which also showed hostile relations.

**TABLE 11:** Confidence interval test.

		90% CI		95% CI		99% CI	
Variables	Coefficient	Low value	High value	Low value	High value	Low value	High value
C	29.186	16.5	41.85	14.07	44.29	9.275	49.09
BS	0.561	–0.04	1.171	–0.165	1.289	–0.396	1.520
FOM	–1.167	–2.59	0.265	–2.876	0.541	–3.419	1.084
NOLS	–0.138	–0.24	–0.027	–0.270	–0.005	–0.312	0.036
CSR	0.032	0.012	0.213	0.002	0.321	0.001	0.421
ROA	–3.010	–8.94	2.918	–10.08	4.062	–12.33	6.30

## Discussion

The study results have revealed that banking size positively relates to banking performance, but the number of large shareholders is negatively related with banking performance. These results are in line with the past studies of Halkos et al. (2016), which showed the banks’ ability to attain maximum output with the minimum quantity of input. These studies depicted the significant importance of banking performance in the banking sector. It has been elaborated by these studies that the improvement in the rate of return of assets accelerates banking institutions’ operational and economic performance. The studies also approved the results of Tsionas and Mamatzakis (2017), which showed the importance of more ROA in attaining superior operational and economic performance. These studies recommended the management of banking and financial institutions to devise their strategies, technology, procedures, and combination of factors in such a way as to attain optimal output by employing minimum quantity of available input. Moreover, the results have indicated that the frequency of meetings is linked with banking and financial sectors’ operational and economic performance. These results were approved by the studies of Pruteanu-Podpiera et al. (2016), according to which the frequency of meetings have a negative association with the performance rate of banking and financial institutions as it motivated them to improve their service which meant they met the customers’ needs and demands. It is suggested by these studies that both the frequency of meetings and financial institutions among different enterprises in other economic industries affects banking performance. The studies also approved results of Jayakumar et al. (2018), which also revealed that the large number of shareholders and frequency meetings negatively affect the banking sector’s operational performance and makes them grow with an increasing rate. These studies elaborate that the large number of shareholders results in improved services as the banks try to have complete information about the changing market trends and customers’ requirements and adapt their activities to match these shifts and requirements. Moreover, the study results have indicated that the bank size had considerable influence on operational and economic performance. These results are approved by a previous study by Nikolaou et al. (2015), which threw light on the fact that the size of a bank may have severe impacts on banking performance while others are less severe. These studies are also in accordance with the past studies of Lehkonen and Heimonen (2015), which also proved the size of a bank increased the banks’ performance.

## Conclusion

This research paper analyzed the consequences of entrepreneurship performance and CSR with innovation on a bank’s financial performance. This research collected a set of ratings to mirror the efficiency of banks’ specific entrepreneurship practices and a set of performance indicators to obtain this objective. The research investigated the relationship between this group of variables. The results showed that differences in entrepreneurship practices are reflected in banks’ actual performance. According to the empirical analysis results, American best-performing firms in terms of entrepreneurship were an example of this latter scenario. The quick score takes the highest-scoring and lowest-scoring firms as a benchmark and evaluates the others subsequently. The negative relationship between the analyzed variables could be due to the reason that best-performer was taken as a benchmark of entrepreneurship effectiveness falling within the second interval, the one in which an increase in entrepreneurship effectiveness decreases performance rather than boosting it. Agency theory predicts that entrepreneur measures foster firm performance by optimizing agency costs and reducing capital waste.

### Limitations

The relationship between the variables has not been consistent throughout all the analyzed samples. The theoretical paradigm used to establish a linkage between the analyzed elements was the agency theory. Only five countries were selected for the data. The analysis provided useful insights to the long-debated question regarding the relevance of entrepreneurship.

### Implication

Findings of the present study suggested that it was not always the case. Extreme strictness in entrepreneurial practices can decrease a bank’s performance. Results concluded that there was a significant positive relation between entrepreneurship, CSR, and innovation on a bank’s financial performance. Government plays a vital role in every field, such as corporations, firms, industries, and the banking sectors Entrepreneurships include the board of directors’ activities, the working activities of external and internal auditors, and the practices of shareholders including common and individual shareholders. This evidence suggested that enhancing entrepreneurship is not always the optimal choice as controls entail costs that may impact negatively on the firm’s results. Instead, entrepreneurship structure should be designed to counterbalance the positive and negative effects associated with it. This is the strategy that leads to the best possible outcome.

## Data Availability Statement

The raw data supporting the conclusions of this article will be made available by the authors, without undue reservation.

## Author Contributions

JW made the conceptual framework. RX made the literature review. MH made the methodology. AS analyzed the data. FA made the discussion. JK made the conclusion. All authors contributed to the article and approved the submitted version.

## Conflict of Interest

The authors declare that the research was conducted in the absence of any commercial or financial relationships that could be construed as a potential conflict of interest.

## Publisher’s Note

All claims expressed in this article are solely those of the authors and do not necessarily represent those of their affiliated organizations, or those of the publisher, the editors and the reviewers. Any product that may be evaluated in this article, or claim that may be made by its manufacturer, is not guaranteed or endorsed by the publisher.
